# Assessment of synthetic MRI to distinguish Warthin’s tumor from pleomorphic adenoma in the parotid gland: comparison of two methods of positioning the region of interest for synthetic relaxometry measurement

**DOI:** 10.3389/fonc.2024.1446736

**Published:** 2024-10-04

**Authors:** Jiabin Sun, Xinping Kuai, Dawei Huang, Xinghua Ji, Chuanhai Jia, Shengyu Wang

**Affiliations:** ^1^ Department of Radiology, Changshu No.2 People’s Hospital, the Fifth Affiliated Clinical Medical College of Yangzhou University, Changshu, Jiangsu, China; ^2^ Department of Radiology, Shanghai Municipal Hospital of Traditional Chinese Medicine, Shanghai University of Traditional Chinese Medicine, Shanghai, China; ^3^ Department of Stomatology, Changshu No.2 People’s Hospital, the Fifth Affiliated Clinical Medical College of Yangzhou University, Changshu, Jiangsu, China; ^4^ Department of Radiology, Ruijin Hospital, shanghai Jiao Tong University School of Medicine, Jiading, Shanghai, China

**Keywords:** parotid neoplasms, pleomorphic adenomas, Warthin’s tumor, magnetic resonance imaging, synthetic MRI, magnetic resonance image compilation

## Abstract

**Purpose:**

To assess the diagnostic potential of the synthetic MRI (SyMRI) for differentiating Warthin’s tumors (WT) from pleomorphic adenomas (PA).

**Materials and methods:**

Forty-nine individuals with parotid gland tumors (PA, n = 23; WT, n = 26) were recruited. Using two distinct regions of interest (ROI), SyMRI quantitative parameters of lesions were calculated, including mean and standard deviation (T1, T2, PD, T1sd, T2sd, and PDsd). Meanwhile, T1ratio, T2ratio, and PDratio (lesion/masseter muscle) were calculated based on the mean SyMRI quantitative parameters of masseter muscle (T1, T2, PD). Using the independent samples *t* test, we compared PA and WT parameters, while comparing the areas under the curve (AUC) using the DeLong’s test. A multi-parameter SyMRI model was constructed using logistic regression analysis.

**Results:**

In PA, the T1, T1sd, T2, PD, T1ratio, T2ratio, and PDratio derived from full and partial lesion ROIs were significantly higher than in WT. According to the receiver operating curve analysis, the AUC of the quantitative parameters derived from full-lesion and partial-lesion ROIs ranged from 0.722 to 0.983 for differentiating PA from WT. T1 values derived from partial-lesion ROI delineation demonstrated the best diagnostic performance among all single parameters, achieving an AUC of 0.983. Using 1322 ms as a cutoff value, the sensitivity, specificity, and accuracy were 88.46%, 100% and 93.88%, respectively.

**Conclusion:**

The SyMRI-derived quantitative parameters demonstrated excellent performance for discriminating PA from WT in the parotid gland.

## Introduction

Pleomorphic adenomas (PA) and Warthin’s tumors (WT) are the most common benign tumors, accounting for more than 70% of the neoplasms in the parotid gland (1, 2). Although PA and WT are benign tumors, they differ in biological behavior and prognosis (2, 3). Unlike WT, PA presents a 1.8% to 6.2% risk of malignant transformation (4, 5). For WT, due to the intact fibrous capsule, enucleation of the tumor alone is sufficient, while for PA, extensive field removal of the tissue in the parotid bed is recommended due to the risk of recurrence or disruption to the capsule (6, 7). Therefore, an accurate preoperative diagnosis of PA and WT is critically necessary for determining the optimal treatment strategy.

Various imaging modalities play essential roles in the diagnosis and management of parotid gland tumors (3, 7). Among these methods, conventional magnetic resonance imaging (MRI) provides information regarding tumor size, position, and relationship to adjacent structures. However, due to variability in the histological component of PA, conventional MRI is insufficient to distinguish PA from WT (8). Quantitative MRI techniques such as diffusion-weighted imaging and dynamic contrast-enhanced MRI (DCE-MRI) can complement physiological and functional information to distinguish subtypes of parotid neoplasms (9, 10). However, the apparent diffusion coefficient (ADC) value of WT overlaps with that of malignant tumors, and ADC alone cannot accurately distinguish benign and malignant parotid tumors (4, 11). In addition, in the case of DCE-MRI, there is a disadvantage due to the risk of serious adverse events and overlapping time-intensity curve pattern between PA and WT (12). Recently, in order to overcome the limited discriminative performance of conventional MRI and other imaging techniques, radiomics has been applied in differentiating various types of parotid tumors (13). Radiomics-based techniques, however, have a number of drawbacks, including limited generalizability of quantitative features due to low sample sizes, single-center studies, and different imaging equipment and radiomics models. Accordingly, novel approaches are required to improve the ability of differential diagnosis of PA from WT.

Synthetic MR imaging, also called magnetic resonance image compilation (MAGiC), can quantitatively measure multiple physical properties based on longitudinal relaxation time (T1), transverse relaxation time (T2) and proton density (PD) parameters (14). Unlike conventional and functional MRI techniques, which are influenced by scan parameters and biophysical model for data postprocessing, the quantitative SyMRI technique is inherently independent of MRI methods and parameters (15). In previous studies, the SyMRI has been applied in the differential diagnosis of breast tumors, prostate lesions and retropharyngeal lymph node (16–18). To our knowledge, there have been no studies exploring SyMRI’s clinical utility for distinguishing PA from WT. Meanwhile, various regions of interest (ROI) delineations have been used in previous studies, but a standardized approach for ROI placement methods is still lacking (19–21). Whole-volume ROI analysis rather than single-slice ROI delineation may accurately reflect tumor heterogeneity (22). In clinical practice, however, it is time-consuming and difficult to perform such analysis. Furthermore, no studies have evaluated the diagnostic performance of SyMRI in distinguishing between PA and WT using different ROI delineations. Hence, this study aimed to assess the clinical feasibility and diagnostic potential of SyMRI for differentiating PA from WT using two different ROI delineation methods.

## Materials and methods

### Participants

All the subjects who participated in the study completed the informed consent form and completed it uniformly. The project was approved by the Ethics Committee of Changshu No.2 People’s Hospital, the Fifth Affiliated Clinical Medical College of Yangzhou University. From January 2021 to December 2023, the SyMRI examination was performed on 71 consecutive individuals with suspected parotid gland tumors using a 3.0T MRI scanner. Exclusion criteria were as follows (4): (1) imaging artifacts interfering with interpretation, (2) prior history of head or neck disease, (3) small parotid gland tumors less than 10 mm in size, (4) the parotid tumor received biopsy, surgery, chemotherapy or radiotherapy and (5) postoperative histopathologically confirmed non-PA or non-WT. Subsequently, three patients with tiny parotid gland tumors under 10 mm in diameter, four patients with severe dental metal artefacts, and 15 patients with histopathologically proven non-PA or non-WT (one carcinoma ex pleomorphic adenoma, two lymphomas, three mucoepidermoid carcinomas, one adenoid cystic carcinomas, one acinar cell carcinomas, one squamous cell carcinomas, two granulomatous lymphadenitis, two myoepithelioma, one basal cell adenoma, and one benign lymphoepithelial lesion) were excluded from this study. Finally, there were 49 patients enrolled in this study, including 23 patients with PAs and 26 patients with WTs. All patients underwent surgical resection, and complete tumor specimens were histopathologically examined within 1 month after their MR examinations.

### MRI data acquisition

All examinations were performed using a GE Signa Architect 3.0T MRI scanner with a 28-channel head-neck coil. Before contrast was administered, SyMRI sequence in the axial direction was performed. The parameters of the SyMRI were as follows: repetition time (TR) of 4000 ms; echo time (TE) of 19.2/96.3 ms; field of view (FOV) of 24 × 24 cm; number of excitations (NEX): 1; Echo Train Length (ETL): 16; Bandwidth:31.25 kHz; matrix: 320 × 256; number of slices:20; slice thickness: 3 mm; slice gap: 1 mm; acquisition time: 4 min.

### Image analysis

The SyMRI quantitative parameters were post-processed by two radiologists blinded to pathology (rater 1 and rater 2 with more than five and ten years of experience in head and neck imaging, respectively). The ROIs were manually delineated from synthetic T2-weighted images by referencing contrast-enhanced T1WI images. Two ROI delineation methods were used: (1) solid portion within the largest section of the lesion, avoiding necrotic, cystic, hemorrhagic, or apparent vessel components (partial lesion); (2) the largest area of the largest section (full lesion) (4, 18). The mean and standard deviation (sd) for longitudinal relaxation time (T1, T1sd), transverse relaxation time (T2, T2sd), and proton density (PD, PDsd) were calculated for WT and PA. Meanwhile, we delineated a ROI on the unilateral masseter muscle at the same level of the lesion and obtained T1, T2, and PD values (23). T1ratio, T2ratio, and PDratio were calculated as follows: T1ratio = T1 value of lesion/T1 value of masseter muscle; T2ratio = T2 value of lesion/T2 value of masseter muscle; PDratio = PD value of lesion/PD value of masseter muscle (23). Tumor volumes were calculated using the following Eq: V=π×L×W^2^/6, where L is length and W is width of the largest section (24).

### Statistical analysis

Two-way mixed, absolute agreement, single measure intraclass correlation coefficients (ICC) were calculated to assess the reliability of SyMRI quantitative parameters between two raters (25). Normal distribution and homogeneity of variance were assessed using the Kolmogorov–Smirnov test and Levene’s test, respectively.PA and WT quantitative parameters were compared through independent sample *t*-tests and categorical variables through chi-squared tests. We plotted receiver operating characteristic curves (ROCs), calculated area under the curve (AUC), optimal threshold values, accuracy, sensitivity, specificity, positive predictive value (PPV), and negative predictive value. Multiparameter diagnostic models were constructed using binary logistic regression analysis (forward stepwise selection model) that included significant parameters with *P* < 0.05. To compare the differences between the ROC curves, DeLong’s test was performed. All statistical analyses were performed with R 4.1.3 and MedCalc software (version 15.2.2) using *P*-values below 0.05.

## Results

As shown in **Table 1**, 49 patients were enrolled in this study, including 23 patients with PA and 26 with WT. No statistical difference was found in tumor volume between the two entities (*P* > 0.05). However, there were statistically significant differences in age and gender ratio between PA and WT (*P* < 0.05). ​In this study, WT showed a predilection for older males.

**Table 1:** Characteristics of patients with PA and WT.

Variable	PA	WT	*P* value
**Age (years)**	48.74 ± 13.88	67.54 ± 7.44	0.005
Gender			
male	12	23	0.005
female	11	3	
**Tumor volume (cm^3^)**	5.72 ± 8.39	6.89 ± 4.93	0.294

Please note that PA stands for pleomorphic adenoma, and WT stands for Warthin’s tumor. Data are expressed as mean ± SD, or number.

### Assessment of SyMRI inter-rater reliability

The ICCs for the quantitative parameters of SyMRI are all above 0.85, indicating excellent inter-rater agreement (**Table 2**).

**Table 2:** Assessment of SyMRI inter-rater reliability.

Parameter	Inter-rater	
	ICC	95%CI
Full-lesion ROI		
T1	0.993	0.988-0.996
T1sd	0.987	0.977-0.993
T2	0.992	0.986-0.996
T2sd	0.997	0.994-0.998
PD	0.988	0.980-0.993
PDsd	0.980	0.965-0.989
T1ratio	0.980	0.964-0.989
T2ratio	0.990	0.982-0.994
PDratio	0.969	0.937-0.984
Partial-lesion ROI		
T1	0.996	0.992-0.998
T1sd	0.988	0.978-0.993
T2	0.998	0.997-0.999
T2sd	0.993	0.988-0.996
PD	0.980	0.964-0.988
PDsd	0.957	0.925-0.975
T1ratio	0.982	0.968-0.990
T2ratio	0.976	0.959-0.987
PDratio	0.960	0.928-0.977
Masseter muscle		
T1	0.914	0.853-0.950
T2	0.887	0.807-0.935
PD	0.962	0.918-0.981

Please note that T1 stands for longitudinal relaxation time, T2 stands for transverse relaxation time, PD stands for proton density, T1sd stands for standard deviation of the T1 measurements, T2sd stands for standard deviation of the T2 measurements, PDsd stands for standard deviation of the PD measurements, ICC stands for intraclass correlation, and 95%CI stands for 95% Confidence Interval.

### SyMRI quantitative parameters for PA and WT

**Figure 1:**
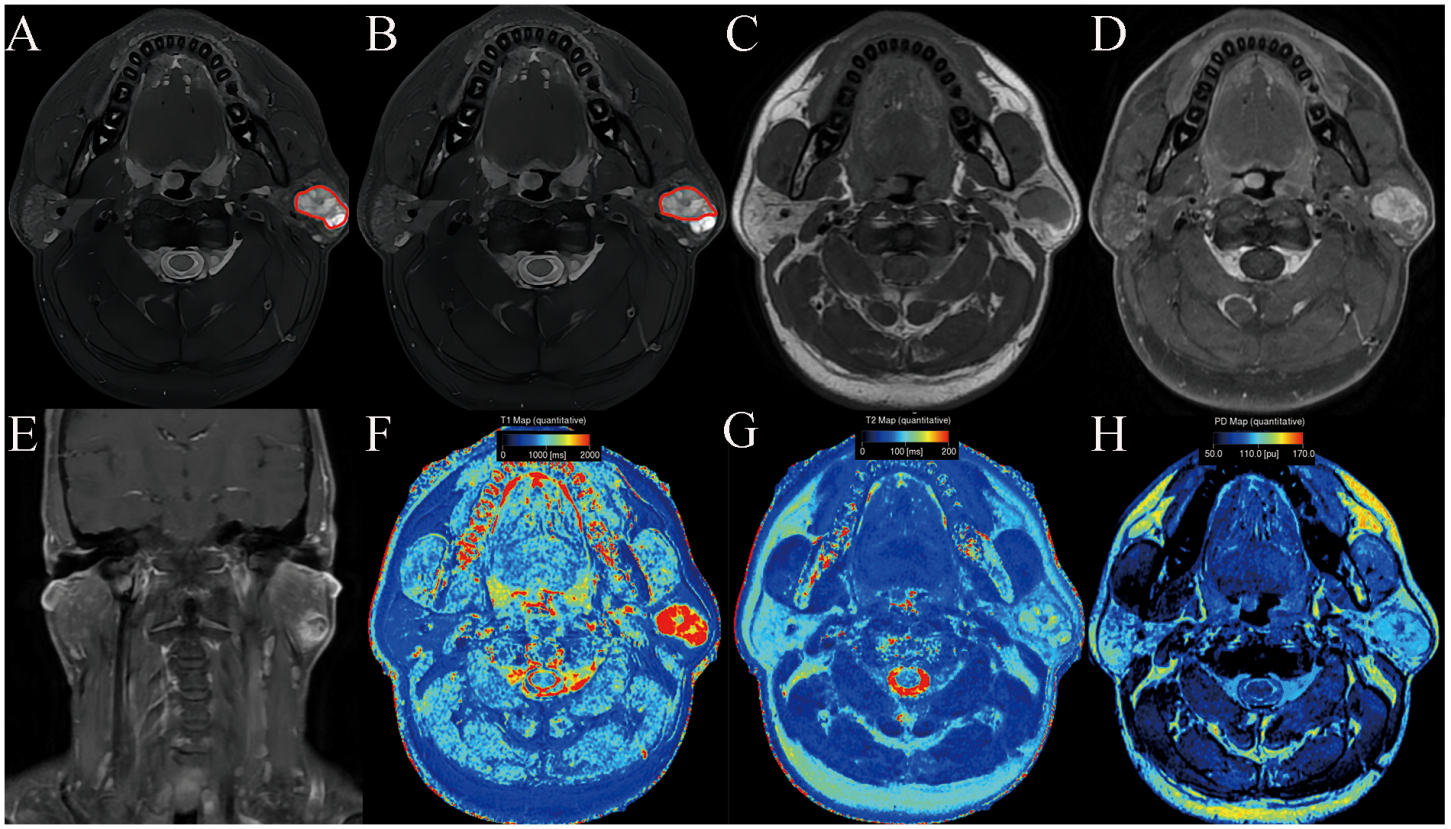
Pleomorphic adenoma of the parotid gland in a 42-year-old male subject. The ROI delineation methods included the largest area of the largest section (full lesion, **A**) and the solid portion within the largest section (partial lesion, **B**). On conventional MRI, this mass showed hyperintense on T2WI **(A, B)**, a well-defined border **(C)**, and marked enhancement on contrast enhanced T1WI **(D, E)**. In comparison with WT, it demonstrated significantly higher SyMRI-derived quantitative parameters for partial-lesion ROI (T1: 2077 ms; T1sd: 529 ms; T2: 90 ms; T2sd: 13 ms; PD:99 pu; PDsd: 8.4 pu) on axial T1 mapping **(F)**, T2 mapping **(G)**, and PD mapping **(H)**.

**Figure 2:**
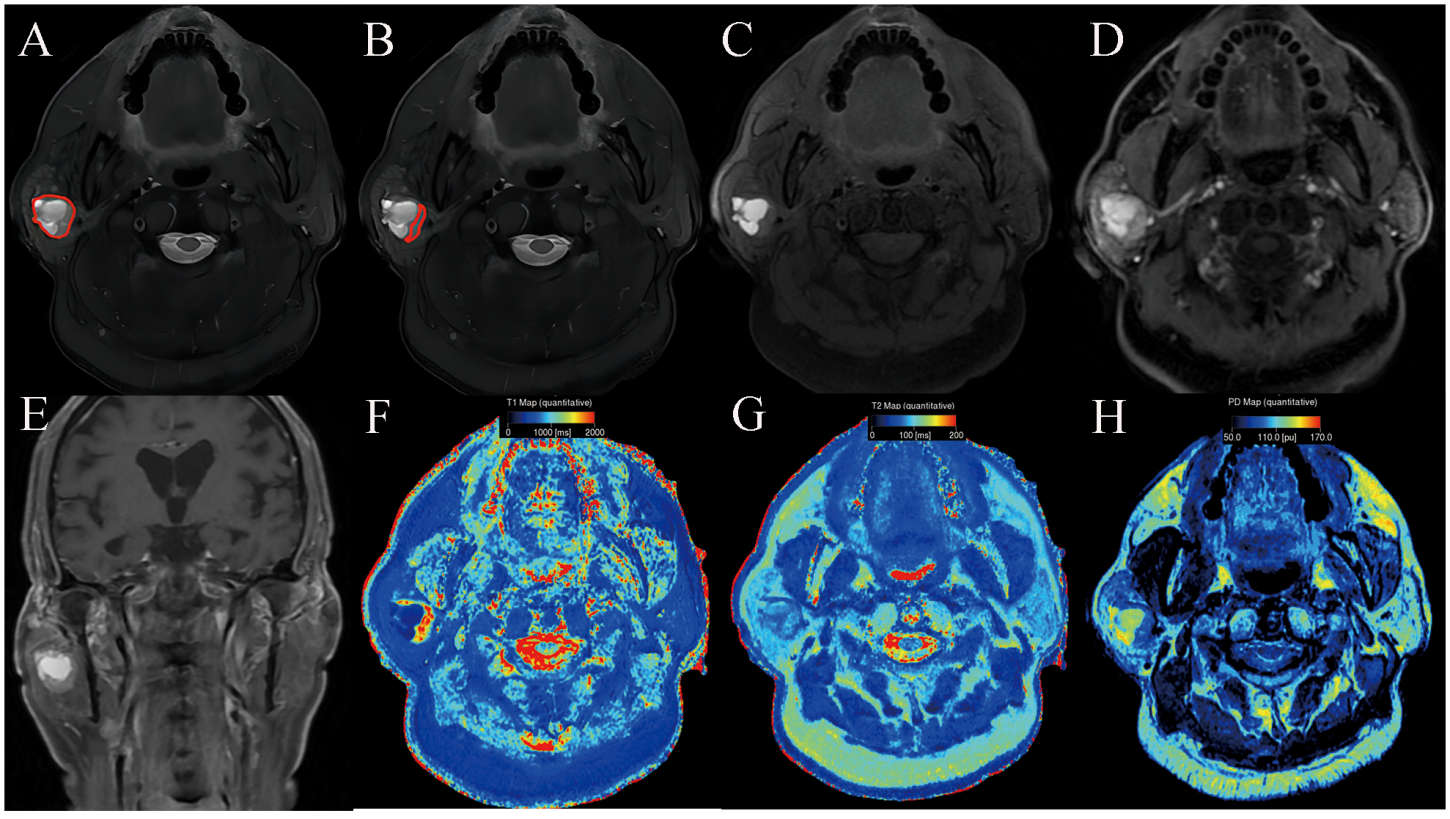
Warthin’s tumor of the parotid gland in a 77-year-old male subject. The ROI delineation methods included the largest area of the largest section (full lesion, **A**) and the solid portion within the largest section (partial lesion, **B**). On conventional MRI, the solid component shows moderate signal and cyst/necrosis shows hyperintense on T2WI **(A, B)**, a well-defined border **(C)**, and moderate enhancement on contrast enhanced T1WI **(D, E)**. In comparison with PA, it demonstrated significantly lower SyMRI-derived quantitative parameters for partial-lesion ROI (T1: 1723 ms; T1sd: 269 ms; T2: 79 ms; T2sd: 7 ms; PD: 86.4 pu; PDsd: 6.1 pu) on axial T1 mapping **(F)**, T2 mapping **(G)**, and PD mapping **(H)**.

**Table 3** summarizes SyMRI’s quantitative parameters for PA and WT. The T1, T1sd, T2, PD, T1ratio, T2ratio, and PDratio of PA derived from full-lesion ROI were significantly higher than those of WT (*P* < 0.05). The T1, T1sd, T2, T2sd, PD, PDsd, T1ratio, T2ratio, and PDratio of PA derived from partial-lesion ROI were significantly higher than those of WT (*P* < 0.05) (**Figures 1**, **2**). In terms of T2sd and PDsd derived from full-lesion ROIs, there were no significant differences between PA and WT (*P* > 0.05). The T1, T2, and PD values measured on the masseter muscle did not differ significantly between the two types of lesions (*P* > 0.05).

**Table 3:** Comparison of quantitative parameters between PA and WT based on full and partial lesion ROIs.

Parameters	PA	WT	*P* value
Full-lesion ROI			
T1	2032.70 ± 486.06	1126.46 ± 229.11	0.000*
T1sd	573.83 ± 230.98	339.62 ± 244.51	0.001*
T2	109.87 ± 43.51	72.19 ± 12.44	0.000*
T2sd	23.48 ± 31.00	11.88 ± 9.13	0.075
PD	88.66 ± 7.62	80.48 ± 7.38	0.000*
PDsd	10.73 ± 5.55	8.88 ± 3.58	0.166
T1ratio	2.04 ± 0.49	1.05 ± 0.23	0.000*
T2ratio	2.34 ± 0.87	1.45 ± 0.27	0.000*
PDratio	1.27 ± 0.22	1.11 ± 0.30	0.031*
Partial-lesion ROI			
T1	2011.91 ± 421.23	1135.61 ± 191.36	0.000*
T1sd	521.65 ± 173.69	231.96 ± 168.48	0.000*
T2	102.74 ± 29.42	70.23 ± 7.89	0.000*
T2sd	16.65 ± 15.47	7.04 ± 2.58	0.003*
PD	87.85 ± 7.35	78.67 ± 4.61	0.000*
PDsd	10.54 ± 5.56	7.23 ± 2.40	0.013*
T1ratio	2.02 ± 0.47	1.07 ± 0.26	0.000*
T2ratio	2.20 ± 0.64	1.42 ± 0.22	0.000*
PDratio	1.26 ± 0.21	1.07 ± 0.24	0.006*
Masseter muscle			
T1	1003.65 ± 112.81	1088.12 ± 198.82	0.071
T2	46.87 ± 2.90	50.35 ± 7.77	0.050
PD	71.13 ± 12.21	75.89 ± 13.66	0.207

ROI, region of interest; T1, longitudinal relaxation time; T1sd, standard deviation of the T1 measurements; T2, transverse relaxation time; T2sd, standard deviation of the T2 measurements; PD, proton density; PDsd, standard deviation of the PD measurements; T1ratio = T1 value of lesion/T1 value of masseter muscle; T2ratio = T2 value of lesion/T2 value of masseter muscle; PDratio = PD value of lesion/PD value of masseter muscle. *Statistical difference. Bold value indicates statistical significance.

### Differentiation between PA and WT patients using SyMRI

**Figure 3:**
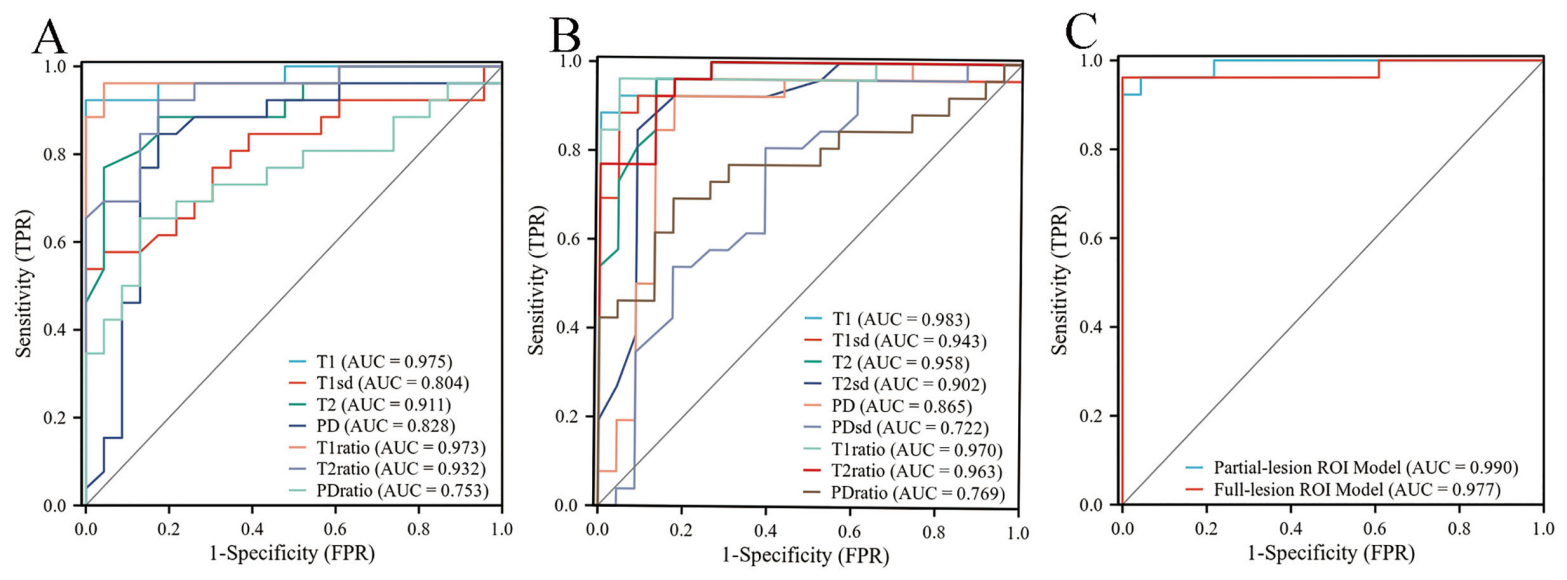
**(A)** ROC curves of quantitative parameters derived from full-lesion ROI delineation for differentiating between PA and WT. **(B)** ROC curves of quantitative parameters derived from partial-lesion ROI delineation for differentiating between PA and WT. **(C)** ROC curves of the combined SyMRI-derived multiple parameters for differentiating between PA and WT. Combined SyMRI-derived multiple parameters showed similar AUCs with these two different ROI delineation methods.

For differentiating PA from WT, the AUCs for these quantitative parameters range from 0.722 to 0.983 based on the ROC analysis. The T1 values obtained from the partial-lesion ROI delineation showed the highest AUC of 0.983 among all single parameters (**Table 4**; **Figures 3A, B**). Using a cutoff value of 1322 ms to discriminate between PA and WT subjects, the T1 values obtained from partial-lesion ROI delineation achieved 88.46% sensitivity, 100% specificity, and 93.88% accuracy, respectively.

**Table 4:** Diagnostic capability of quantitative parameters calculated from full-lesion and partial-lesion ROIs for differentiating PA from WT.

	AUC (95% CI)	Cutoff value	Youden index	Sen (%)	Spe (%)	PPV (%)	NPV (%)	Acc (%)
Full-lesion ROI								
T1	0.975 (0.935-1.000)	1378.5	0.923	92.31	100	100	92.00	95.92
T1sd	0.804 (0.677-0.930)	250	0.538	53.84	100	100	65.71	75.51
T2	0.911 (0.830-0.991)	78.5	0.725	76.92	95.65	95.24	78.57	85.71
PD	0.828 (0.697-0.959)	84.1	0.672	84.61	82.61	84.61	82.61	83.67
T1ratio	0.973 (0.926-1.000)	1.38	0.918	96.15	95.65	96.15	95.65	95.92
T2ratio	0.932 (0.866-0.999)	1.80	0.749	92.31	82.61	85.71	90.47	87.75
PDratio	0.753 (0.611-0.894)	1.07	0.523	65.38	86.96	85.00	68.96	75.51
Combined (SyMRI)	0.977 (0.930-1.000)	0.24	0.961	96.15	100	100	95.83	97.96
Partial-lesion ROI								
T1	0.983 (0.958-1.000)	1322	0.885	88.46	100	100	88.46	93.88
T1sd	0.943 (0.864-1.000)	301.5	0.841	88.46	95.65	95.83	88.00	91.84
T2	0.958 (0.909-1.000)	80.5	0.831	96.15	86.96	89.28	95.24	91.84
T2sd	0.902 (0.807-0.998)	8.5	0.759	84.61	91.30	91.67	84.00	87.75
PD	0.865 (0.747-0.982)	83.75	0.749	92.31	82.61	85.71	90.47	87.75
PDsd	0.722 (0.574-0.871)	8.25	0.416	80.77	60.87	70.00	73.68	71.43
T1ratio	0.970 (0.919-1.000)	1.42	0.918	96.15	95.65	96.15	95.65	95.92
T2ratio	0.963 (0.921-1.000)	1.73	0.793	92.31	86.96	88.89	90.91	89.80
PDratio	0.769 (0.632-0.906)	1.08	0.518	69.23	82.61	81.82	70.37	75.51
Combined (SyMRI)	0.990 (0.971-1.000)	0.26	0.923	92.31	100	100	92.00	95.92

ROI, region of interest; Sen, Sensitivity; Spe, Specificity; PPV, positive predictive value; NPC, negative predictive value; Acc, accuracy; T1, longitudinal relaxation time; T1sd, standard deviation of the T1 measurements; T2, transverse relaxation time; T2sd, standard deviation of the T2 measurements; PD, proton density; PDsd, standard deviation of the PD measurements; T1ratio = T1 value of lesion/T1 value of masseter muscle; T2ratio = T2 value of lesion/T2 value of masseter muscle; PDratio = PD value of lesion/PD value of masseter muscle. Combined (SyMRI) derived from full-lesion ROI: Logit (p = PA) = 6.74×T1ratio+2.37×T2ratio-13.89; Combined (SyMRI) derived from partial-lesion ROI: Logit (p = PA) = 7.68× T1ratio+0.40×T2sd-15.38.

The AUCs for T1, T2, PD, T1ratio, T2ratio and PDratio values did not differ between the partial and full lesion ROI delineation methods (all *P* > 0.05). Combined SyMRI-derived multiple parameters resulted in similar AUCs when delineating ROIs with these two different approaches (*P* > 0.05) (**Figure 3C**). The AUCs for T1sd, T2sd, and PDsd values differed significantly between partial and full lesion ROI delineations (all *P* < 0.05).

## Discussion

In this study, based on full and partial lesion ROIs, we observed that T1, T1sd, T2, PD, T1ratio, T2ratio, and PDratio were higher in PA than in WT. Moreover, the T2sd and PDsd of PA derived from partial-lesion ROI were significantly higher than those of WT. No significant differences, however, were found in the T2sd and PDsd derived from full-lesion ROI between the PA and WT groups. We also demonstrated the ability of SyMRI parameters to discriminate between subjects with PA and those with WT. As far as we know, this study is the first to investigate the quantitative parameters of SyMRI and assess its diagnostic power for PA and WT individuals.

A previous study using T1 mapping in parotid gland tumors has demonstrated that the mean T1 relaxation time is higher in PA than in WT (26). Similarly, we revealed that the T1 value of PA was significantly higher than that of WT using SyMRI, with either partial or full lesion ROI delineation approaches. The T1 values obtained from the partial-lesion ROI delineation showed the highest AUC of 0.983 among all single parameters. Using a cutoff value of 1322 ms to discriminate between PA and WT subjects, the T1 values obtained from partial-lesion ROI delineation achieved 88.46% sensitivity, 100% specificity, and 93.88% accuracy, respectively. T1 value is influenced by water content, binding with macromolecules (water mobility), and cell content (27, 28). The higher T1 values of PA were attributed to the tiny nuclei of the epithelial and mesenchymal cells, low cell and neovascular densities, abundant stroma, large extracellular spaces, and excessive free water content in these tumors (29). The lower T1 values of WT may be attributed to the abundance of lymphocytes and lymph stromal cells, high microvascular density and cellular-stromal grade, low free water content, relatively small extracellular space, and limited diffusion of water molecules (29). This suggests that T1 values may be valuable to distinguish PA from WT. To our knowledge, there are no studies published in the literature comparing PA and WT based on the standard deviation of T1 measurement (T1sd) value and T1 value of lesion/T1 value of masseter muscle (T1ratio). In this study, PA had a significantly higher T1sd than WT, suggesting that PA has more T1 variability over image pixels compared to WT. At the cellular level, PA is characterized by morphological diversity that includes both epithelial and mesenchymal components, which may lead to higher T1 variability within ROIs. Lastly, we found that T1ratios were significantly higher in PA than in WT, yielding an AUC of 0.973 for full-lesion ROI, and 0.970 for partial-lesion ROI. Therefore, the T1ratio value may also be a valuable quantitative parameter for differentiating PA from WT.

In different tissues, T2 values (quantification of transverse relaxation time) have been identified as excellent and reproducible biomarkers of water content (especially free water molecules). A previous study using T2 mapping in parotid gland tumors has demonstrated that the mean T2 relaxation time is higher in PA than in WT (30). Our previous study found that the solid components of PA mostly showed hyperintense on T2-weighted imaging compared with WT (4). Using SyMRI, either partial or full lesion ROI delineation, we found that PA had a higher T2 value than WT. The similarities in these findings may be explained by the pathological features of PA and WT. This suggests that T2 values may be valuable to distinguish PA from WT. To our knowledge, there are no studies published in the literature comparing PA and WT based on the standard deviation of T2 measurement (T2sd) value and T2 value of lesion/T2 value of masseter muscle (T2ratio). In this study, we found that the T2sd of PA derived from partial-lesion ROI was significantly higher than that of WT. However, no significant difference between the PA and WT groups was confirmed in the T2sd derived from the full lesion ROI. Thus, the T2sd derived from partial-lesion ROI and T2ratio value may be valuable quantitative parameters for differentiating PA from WT.

The PD value, as another magnetic property of the tissue, primarily reflects the water content of tissue (31). A previous study found that the PD value could distinguish benign from malignant retropharyngeal lymph node (18). In our study, we found that PA had higher PD and PDratio values compared to WT. In addition, we found that the PDsd of PA derived from partial-lesion ROI was significantly higher than that of WT. However, no significant difference between the PA and WT groups was confirmed in the PDsd of PA derived from full-lesion ROI. Thus, the PD value, PDsd derived from partial-lesion ROI and PDratio value may be also valuable quantitative parameters for differentiating PA from WT.

In our study, two different ROI approaches, partial or full-lesion ROI, were conducted in parameter measures. No significant differences between partial- and full-lesion ROI delineation approaches were observed in the AUCs of T1, T2, PD, T1ratio, T2ratio, and PDratio values. Meanwhile, combined SyMRI-derived multiple parameters showed similar AUCs for two different ROI delineation approaches. Thus, different types of ROI selection schemes have insignificant effect on the quantitative parameters of SyMRI for distinguishing PA from WT.

Our study had several limitations. Firstly, we revolved only around PA and WT due to low incidence and low sample size of the other parotid tumors. Secondly, this was a single-center study, which limits the generalizability of our findings. So future multicenter studies are needed to confirm our findings. However, the T1, T2, and PD values of MRI are fundamental properties independent of the MRI scanners or scanning parameters at a given field strength, suggesting our findings could be generalized to other hospitals. Finally, future studies should use multiple imaging modalities to detect more sensitive and robust biomarkers for distinguishing PA from WT.

In conclusion, the quantitative parameters of SyMRI were shown to be efficient in distinguishing PA from WT, with T1 values derived from partial lesion ROI delineation demonstrating the best diagnostic performance among all individual parameters. Our findings may have important implications for preoperatively identifying optimal treatment strategies.

## Data Availability

The original contributions presented in the study are included in the article/supplementary material, further inquiries can be directed to the corresponding author/s.
